# 2246. Urine Culture Clinical Decision Support Menu for Reducing Inappropriate Lab Ordering

**DOI:** 10.1093/ofid/ofac492.1864

**Published:** 2022-12-15

**Authors:** Erik S Stensgard, Dimitri M Drekonja, Bobbie Masoud

**Affiliations:** Minneapolis VA Health Care System, Hastings, Minnesota; Minneapolis VA Health Care System, Hastings, Minnesota; Minneapolis VA Health Care System, Hastings, Minnesota

## Abstract

**Background:**

Antibiotic treatment of asymptomatic bacteriuria (ASB) is unnecessary except in pregnant women or those undergoing invasive urologic procedures. Unnecessary treatment of ASB is an important driver of inappropriate antimicrobial use (IAU), leading to antimicrobial resistance, *Clostridioides difficile* infection, adverse drug events, and increased costs. Because ASB requires detection to be treated, unnecessary urine cultures (UC) are a key cause of IAU. Strong evidence supports not obtaining a UC from asymptomatic patients.

**Methods:**

To reduce unnecessary UC orders at the Minneapolis Veterans Affairs Health Care System (MVAHCS), UC orders within the electronic health record (EHR) were redirected to a UC clinical decision support (CDS) menu (Figure 1). Selection of an indication from the defined list is required to place a UC order and provides tracking. UC order data was obtained from the Corporate Data Warehouse (CDW), the VA’s data program. Patient bed days were collected from a CDW dashboard developed by the Iowa City Veteran’s Affairs Health Care System. Data was visualized using Microsoft Power BI^TM^ platform.

**FIGURE 1.**
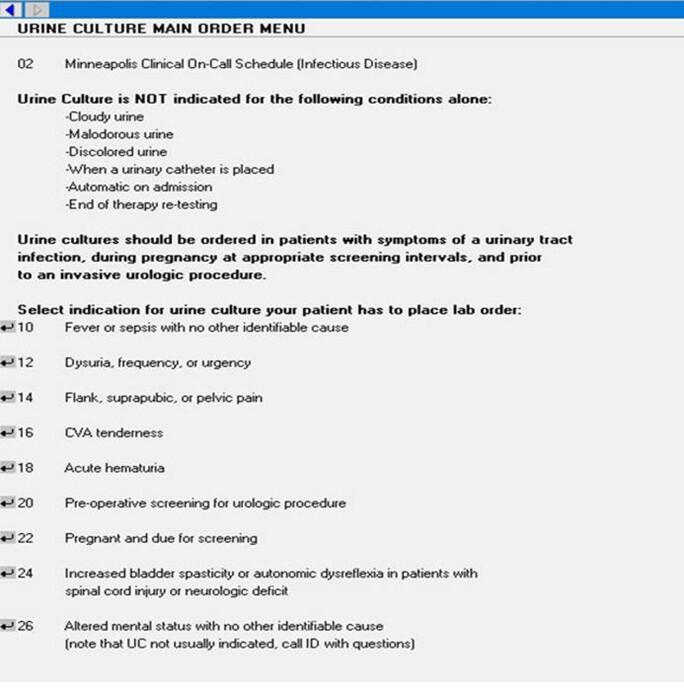
Urine culture clinical decision support menu. Urine culture orders are items numbered 10-26.

**Results:**

The UC CDS menu was implemented at the MVAHCS in September 2020. UC orders from 16 months prior to implementation (9/1/2018 - 12/31/2019) was compared to orders 16 months after implementation (9/1/2020 - 12/31/2021). Data from 1/1/2020 - 8/31/2020 was not included due to atypical patient care patterns during the COVID-19 pandemic.^4^ The monthly number of UC orders after implementation significantly decreased from an average of 765 to 564, a 26.3% reduction (*P* < .001; 2-sided t-test) (Figure 2). The average patient bed days prior to and following implementation was not significantly different (Figure 3). Most UC orders came from the UC CDS menu (8103, 89.8%) compared to orders placed from other order menus or directly from the drug file (920, 10.2%). The most common indication selected was dysuria, frequency, and urgency (4050, 44.9%) followed by fever or sepsis (1230, 13.6%) then pre-operative urologic screening (1056, 11.7%) (Figure 4).

**FIGURE 2.**
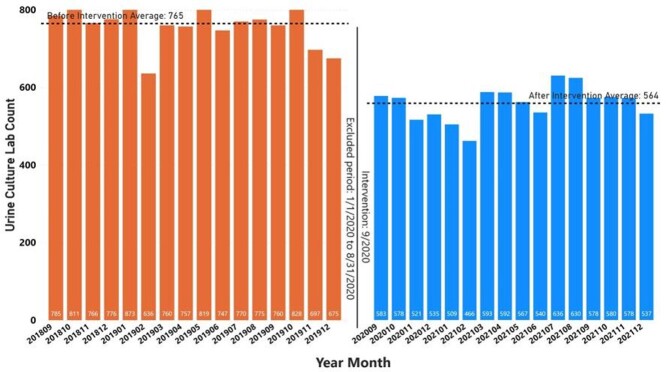
Urine culture lab count before and after urine culture clinical decision support menu intervention.

**FIGURE 3.**
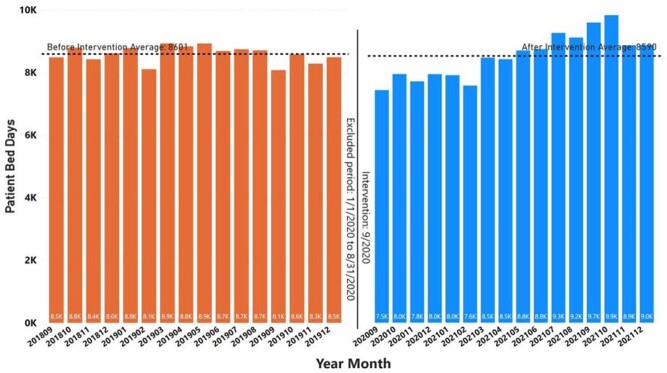
Patient bed days before and after urine culture clinical decision support menu intervention.

**FIGURE 4.**
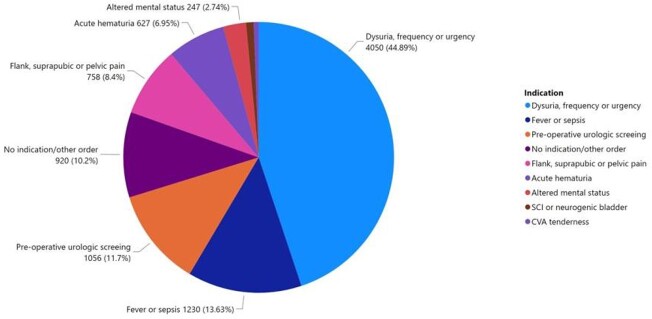
Urine culture orders by indication. Non-intervention labs are listed as no indication/other order.

**Conclusion:**

Implementation of a UC CDS menu within the MVAHCS EHR resulted in significantly fewer UC orders. Most UC orders had an appropriate indication suggesting the decrease was primarily due to preventing unnecessary UC orders.

**Disclosures:**

**All Authors**: No reported disclosures.

